# The influence of a gluten-free diet on health-related quality of life in individuals with celiac disease

**DOI:** 10.1186/s12876-021-01908-0

**Published:** 2021-08-25

**Authors:** Fahdah F. Al-sunaid, Maha M. Al-homidi, Rawan M. Al-qahtani, Reema A. Al-ashwal, Ghada A. Mudhish, Mahitab A. Hanbazaza, Abeer S. Al-zaben

**Affiliations:** 1 Clinical Nutrition Program, Department of Health Sciences, College of Health and Rehabilitation Sciences, Princess Nourah Bint Abdulrahman University (PNU), PO Box 84428, Riyadh, Kingdom of Saudi Arabia; 2 Department of Food and Nutrition, Faculty of Human Sciences and Design, King Abdulaziz University (KAU), Jeddah, Kingdom of Saudi Arabia

**Keywords:** Celiac disease, Health-related quality of life, Gluten-free diet, Food insecurity

## Abstract

**Background:**

Adherence to a gluten-free diet (GFD) and food insecurity (FI) may influence health-related quality of life (HRQOL) in individuals with celiac disease (CD). This study aimed to investigate the association between adherence to a GFD, FI, and HRQOL in individuals with CD.

**Methods:**

This cross-sectional study included 97 adults (mean age: 34 ± 9 years) diagnosed with CD. The participants were on a GFD for more than 6 months. Sociodemographic characteristics and medical history were assessed. Adherence to a GFD, FI, and HRQOL were assessed using validated questionnaires.

**Results:**

Most participants (73%) adhered to a GFD, and 62% were experiencing FI. Individuals with CD faced difficulty in accessing GF foods due to the high cost (90%) and limited availability (79%). The mean overall HRQOL score was 60. Scores on the physical and mental health domains were 69 and 47, respectively. Adherence to a GFD was significantly associated with FI (*P* = 0.02), while there was no association between adherence to a GFD and HRQOL measures (*P* > 0.05). Participants facing FI had lower scores in emotional well-being and mental health domains, and overall HRQOL (*P* < 0.05).

**Conclusions:**

The findings of the present study demonstrate that FI influences adherence to a GFD, and that FI is associated with HRQOL in terms of both emotional well-being and mental health.

## Background

Celiac disease (CD) is an autoimmune disease that affects the small intestine, whereby gluten ingestion leads to the destruction of enterocytic villi in affected patients [1]. The primary genes involved in CD development are HLA DQ2 and HLA DQ8 [1, 2]. CD occurs in 1 to 2 individuals per 100 worldwide; however, systemic reviews reported that the prevalence of CD in Saudi Arabia is higher (approximately 3%) [3, 4]. Individuals with CD may experience a broad variety of gastrointestinal and malabsorption symptoms (classical CD) or extraintestinal symptoms (non-classical CD) [5]. The only available treatment for CD is lifelong adherence to a gluten-free diet (GFD) [6]. Adherence to a GFD refers to strict elimination of products that contain gluten such as wheat, barley, rye, and foods that are derivatives of grains (e.g., semolina, durum, spelt, triticale, and malt) [6, 7]. In previous studies, the rate of adherence to a GFD varied from 44 to 90% in patients with CD [8, 9]. These studies have highlighted various factors that influence adherence to a GFD, such as avoidance to travel and accepting invitations due to strict requirements of a GFD, limited availability of gluten-free products, duration of disease, age at diagnosis, and region of residence [8–12]. The limited availability of gluten-free products may also influence food security among patients with CD attempting to adhere to a GFD [8].

Food insecurity (FI) is a social and economic condition that leads to restricted access to nutritious food [13]. The concept of FI includes food affordability, availability, and the acceptability of foods within one’s culture [14]. Food security is achieved when all individuals have financial, physical, and social access to adequate food for maintaining nutritional requirements and a healthy life at any given time [15]. Patients with CD may face difficulty due to the higher cost and limited availability of gluten-free foods, which may in turn affect their emotional state and health-related quality of life (HRQOL) [16–19]. Decreased physical health due to gastrointestinal symptoms may also impact HRQOL in patients with CD [20]. In addition, there is strong evidence that CD is associated with depression, anxiety, social pressure, and difficulties in daily social relations due to the nature of the disease and/or adherence to a GFD [19]. Finally, economic status may also influence social and emotional states among patients with CD, leading to lower HRQOL in these domains [21].

Although CD and its potential complications may negatively affect HRQOL, information regarding the association between adherence to a GFD, FI, and HRQOL is limited in Saudi Arabia. Therefore, this study aimed to assess the association between adherence to a GFD, FI, and HRQOL in a cohort of individuals with CD living in Saudi Arabia.

## Methods

### Study design and participants

This pilot cross-sectional study included 97 adult individuals with CD living in Saudi Arabia. Participants were recruited from the Saudi Celiac Association and an online support group. This approach was similar to that of the other study conducted in the UK [22]. The Saudi Celiac Association distributed the survey to members who have CD and participants who were interested in this study had contacted us. After explaining the study to the participants via phone, the participants agreed to enroll in this study. The link of the consent form and the survey was distributed via What’s App messages. Inclusion criteria were as follows: age 18 to 65 years, residence in Saudi Arabia, and diagnosis of CD (biopsy and/or serology) more than 6 months prior to study enrolment to guarantee adequate GFD knowledge. Participants with any other condition that can affect HRQOL (e.g., multiple food allergies, diabetes mellitus type 1, inflammatory bowel disease, hepatitis C, multiple sclerosis, coronary artery disease, end-stage renal disease, stroke, kidney transplant, and hemodialysis treatment) were excluded. All subjects gave their informed consent for inclusion before they participated in the study. The study was conducted in accordance with the Declaration of Helsinki, and the protocol was approved by the Ethics Committee of Princess Nourah bint Abdulrahman University (H-01-R-059), IRB log number (20–0011).

### Sociodemographic characteristics and medical history

Data related to sociodemographic factors were collected including age, gender, nationality, education level, marital status, area of residence, and monthly family income. Anthropometric information including weight and height was self-reported. Medical history was assessed based on the family history of CD, age at CD diagnosis, comorbid diseases such as diabetes mellitus, food allergies, or food intolerance, and whether patients were symptomatic (abdominal bloating, diarrhea, headache, and flatulence) or asymptomatic [22].

### Adherence to a GFD

Adherence to a GFD was assessed using the validated Biagi questionnaire, which is a simple survey consisting of four questions [23]. The questionnaire was translated to Arabic and back translated. After translation, the questionnaire was sent to the participants in Arabic. Total scores in this questionnaire range from 0 to 5. Scores of 0–1 indicate that the patient does not strictly adhere to a GFD, while a score of 2 indicates that the patient follows a GFD but requires further education based on a question asking the participants if they do read and check food labels on packaged food. Scores of 3–5 reflect strict adherence to a GFD [23].

### FI status

FI status was assessed using the Arabic Version of the Food Insecurity Experience Scale Survey Module (FIES-SM) [21]. The FIES-SM has been validated in Middle Eastern countries [24–26]. The FIES-SM contains eight items related to food consumption over the last 12 months. The response to these questions was yes or no (1 vs. 0). Final scores range from 0 to 8 based on the affirmative response. Scores 0–1 indicate food security, 2–3 indicate mild FI (worrying about the ability to obtain food), 4–6 moderate FI, and 7–8 indicate severe FI (hunger) [27]. We included two additional questions adapted from previous studies [16, 28]. The first question assessed the accessibility of gluten-free processed foods (gluten-free: bread, pasta, breakfast cereal, flour, and snacks) in grocery stores in Saudi Arabia. Three possible answers were included: very accessible, somewhat accessible, and not accessible. The second question assessed the seven challenges individuals with CD face to afford a GFD: high price, distance to grocery stores, physical disabilities, not available, limited variety of items, low quality of GFD, or/and not provided in the hospitals.

### Assessment of HRQOL

HRQOL was assessed using the 36-Item Short Form Survey (SF-36), which includes 36 questions across eight scales: physical functioning, role limitations due to physical health, pain, general health, role limitations due to emotional problems, energy/fatigue, emotional well-being, and social functioning. The items were further clustered into two summary domains (physical health and mental health), and the overall HRQOL was calculated based on the average of physical and mental health domains. All items were scored from 0 to 100, with higher scores indicating better HRQOL [29–31]. SF-36 was validated and used previously in Saudi population [32].

### Statistical analysis

Data were analyzed using the Statistical Package for the Social Sciences (SPSS) version 25 (2017). *P* values < 0.05 were considered statistically significant. Normally distributed variables are presented as mean (± SD), while skewed variables are presented as median and interquartile range (IQR). Due to the small sample size of some groups when assessing education levels and areas of residence, patients were classified into two groups when assessing the association between these factors and adherence to GFD, FI, and HRQOL. For analyses of education levels, we compared patients who had obtained at least a bachelor’s degree with those who had not. For residential analyses, we compared patients residing in central regions with those residing in non-central regions (i.e., south, east, west, and north regions). Mann–Whitney U test was used to compare non-normally distributed variables, while the independent t-test was used to compare normally distributed variables. Chi-square test was used to compare categorical variables. Correlation test was performed to assess the association between the continuous sociodemographic, anthropometric, and medical history variables (age, body mass index (BMI), and duration of the disease), and HRQOL.

## Results

### Sociodemographic characteristics and medical history

One hundred and fifteen participants who were interested in this study were enrolled. One participant who was < 18 years old and 17 participants in whom CD was diagnosed within 6 months prior to enrollment were excluded. Ninety-seven participants met the inclusion criteria and were included in the study. Table 1 displays the sociodemographic characteristics of the included participants. Most participants were female. Underweight status was noted in 13% of participants, while overweight and obese status was noted in 28% and 15% of participants, respectively. Most participants were of Saudi descent, had at least a bachelor’s degree, were married, lived in the central region of the country, and had a monthly income less than 5,000 Saudi Riyals (SAR).

**Table 1:** Sociodemographic characteristics of participants with celiac disease living in Saudi Arabia (n = 97)

Variables	N number (%) or Mean ± Standard division
Age (years)	34 ± 9
Weight (kg)	60 ± 15
Height (cm)	158 ± 8
BMI (kg/m^2^)	24.2 ± 5.3
Gender	
Male	11 (11%)
Female	86 (89%)
Nationality	
Saudi	92 (95%)
Non-Saudi	5 (5%)
Education Level	
Intermediate school	7 (7%)
High school graduate	22 (23%)
Bachelor’s degree	53 (55%)
Diploma	10 (10%)
Higher education (Master and PhD)	5 (5%)
Marital status	
Single	34 (35%)
Married	60 (62%)
Divorced	3 (3%)
Widowed	0 (0%)
Regions of Residence	
Central region	70 (72%)
South region	9 (9%)
East region	6 (6%)
West region	7 (7%)
North region	5 (5%)
Monthly family income	
Less than 5,000 SAR/month	53 (55%)
5,000 to 10,000 SAR/month	21 (21%)
More than 10,000 SAR/month	23 (24%)

Data are presented as mean ± SD or number of participants (%)

BMI, body mass index; SAR, Saudi Riyals; SD, standard deviation

Forty-two participants (43%) had a family history of CD. Symptoms related to CD such as diarrhea, headache, and flatulence appeared in most participants (n = 63; 65%), while only 35% were asymptomatic (n = 34). While the majority of the participants (n = 73, 75%) had CD alone with no other comorbidities, 24 participants had CD with other diseases (lactose intolerance or one food allergy (e.g. egg). The mean age at diagnosis was 28 ± 11 years (range: 5–51 years). The mean duration of following GFD was 6 ± 5 years (range: 1–37 years).

### Adherence to a GFD

Approximately 27% (*n* = 26) of participants were not adhering to a GFD. Among the 71 participants (73%) adhering to a GFD, none exhibited low knowledge. Individuals adhering to a GFD were significantly older (36 ± 9 years) than those not adhering to such a diet (29 ± 7 years) (*P* = 0.001). No significant association was observed between adherence to a GFD and any other sociodemographic factors (weight, height, BMI, educational level, marital status, region of residence, and income) (*P* > 0.05). Moreover, medical history (family history of CD, age at CD diagnosis, related comorbidities, and symptomatic status) was not significantly associated with adherence to a GFD (*P* > 0.05).

### FI status

**Fig. 1:**
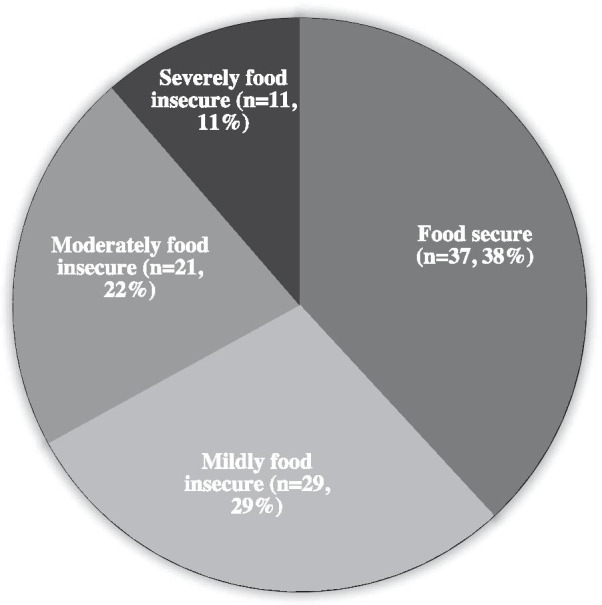
FI status in individuals with CD assessed using the Food Insecurity Experience Scale Survey Module (n = 97)

Figure 1 represents the food insecurity status in individuals with CD assessed using the Food Insecurity Experience Scale Survey Module. Only 38% of participants (*n* = 37) reported food security. Thus, 62% of participants (*n* = 60) faced FI in the present study. Most participants with FI (*n* = 40, 76%) had family incomes below 5,000 SAR/month, while most with food security (*n* = 14, 61%) had incomes over 10,000 SAR/month (*P* = 0.007). Individuals living in the central region of Saudi Arabia were significantly more food secure (*n* = 31, 84%) than patients living in non-central regions (*n* = 6, 16%) (*P* = 0.045). No significant association was observed between FI and sociodemographic characteristics (age, weight, height, BMI, nationality, educational level, or marital status) or medical history (family history of CD, age at CD diagnosis, presence of related comorbidities, symptomatic status) (*P* > 0.05).

**Fig. 2:**
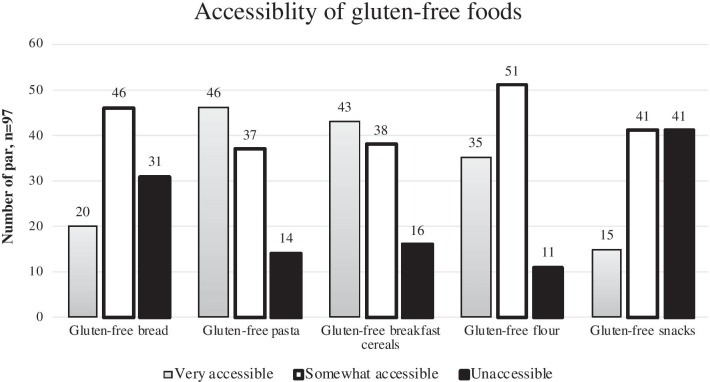
The accessibility of gluten-free processed foods among participants with CD (n = 97). X-axis represents the gluten-free processed foods and Y-axis represents the number of participants

**Fig. 3:**
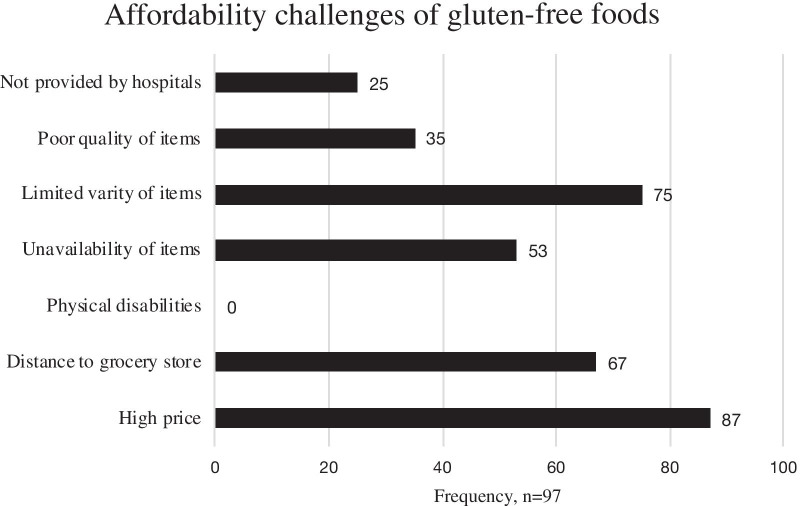
Factors influencing affordability of gluten-free foods in participants with celiac disease (n = 97). X-axis represents the number of participants and Y-axis represents the factors influencing affordability of gluten-free foods

Figure 2 shows the accessibility of gluten-free processed foods among participants with CD. Gluten-free pasta (*n* = 46, 47%) and gluten-free breakfast cereal (*n* = 43, 44%) were the two most accessible foods from grocery stores in Saudi Arabia. Figure 3 illustrates the challenges that participants faced in accessing gluten-free food. Gluten-free foods were not available in the supermarket for more than half of participants (*n* = 53, 55%). Additional challenges to access gluten-free foods included travel, lack of Arabic breads, and limited gluten-free options at restaurants.

### HRQOL

Table 2 displays HRQOL findings in participants with CD. Age (*r* = − 0.256, *P* = 0.012) and BMI (*r* = − 0.221, *P* = 0.035) exhibited significant weak inverse correlations with physical functioning scores. Women scored significantly lower than men on physical functioning (women: median, 75 [IQR, 50–95], men: median, 100 [IQR, 90–100], *P* = 0.001) and emotional well-being scales (women: 54 ± 20, men: 70 ± 18, *P* = 0.014). Participants who had obtained at least a bachelor’s degree scored significantly higher on the emotional well-being scale than individuals who had not attained a bachelor’s degree (bachelor’s degree: 59 ± 20, without a bachelor’s degree: 51 ± 19, *P* = 0.041). Participants living in the central region of the country had significantly higher HRQOL scores than those living in non-central regions on the following domains: role limitations due to physical health problems (central: median, 100 [IQR, 25–100], non-central: median, 25 [IQR, 0–100], *P* = 0.011), energy/fatigue (central: median, 50 [IQR, 39–60], non-central: median, 40 [IQR, 30–50], *P* = 0.016), social functioning (central: median, 63 [IQR, 50–88], non-central: median, 50 [IQR, 38–75], *P* = 0.039), physical health (central: median, 73 [IQR, 50–88], non-central: median, 56 [IQR, 44–78], *P* = 0.033), and mental health (central: median, 50 [IQR, 39–74], non-central: median, 40 [IQR, 26–69], *P* = 0.037). The income was categorized to three categories: < 5,000 SAR/month, 5,000 to 10,000 SAR/month, and > 10,000 SAR/month. Participants with a higher income (> 10,000 SAR/month) scored significantly higher on physical functioning (median, 95 [IQR, 85–100], median, 65 [IQR, 43–75], median, 80 [IQR, 50–100], *P* = 0.000), energy/fatigue (median, 60 [IQR, 50–70], median, 45 [IQR, 30–50], median, 45 [IQR, 35–53], *P* = 0.000), emotional well-being (67 ± 15, 55 ± 18, 52 ± 21, *P* = 0.009), pain (median, 90 [IQR, 68–100], median, 58 [IQR, 45–79], median, 78 [IQR, 55–89], *P* = 0.04), physical health (median, 85 [IQR, 66–89], median, 53 [IQR, 44–74], median, 78 [IQR, 55–89], *P* = 0.003), and mental health domains (median, 72 [IQR, 44–85], median, 45 [IQR, 30–68], median, 45 [IQR, 32–67], *P* = 0.015) than those with lower incomes of 5,000–10,000 SAR or < 5,000 SAR per month, respectively. Marital status and medical history were not significantly associated with HRQOL (*P* > 0.05).

**Table 2:** Health-related quality of life based on Short Form 36 findings in participants with celiac disease living in Saudi Arabia (n = 97)

SF-36	Patients with CD (*n* = 97)	Adherence to GFD (*n* = 71)	Non-adherence to GFD (*n* = 26)	*P* value^a^	Food secure (*n* = 37)	Food insecure (*n* = 60)
Physical functioning^b^	80 (58–100)	80 (50–100)	)8765–100)	0.435	85 (65–100)	75 (51–95)
Role limitation due to physical health problems^b^	75 (13–100)	75 (25–100)	75 (0–100)	0.851	100 (25–100)	63 (0–100)
Role limitation due to emotional problems^b^	33 (0–100)	67 (0–100)	0 (0–100)	0.083	67 (0–100)	0)0–100)
Energy/ fatigue^b^	50 (35–60)	50 (35–60)	45 (40–50)	0.344	55 (38–60)	45 (35–54)
Emotional well-being^c^	56 ± 20	56 ± 21	55 ± 17	0.734	62 ± 20	52 ± 19
Social functioning^b^	63 (38–88)	63 (38–88)	63 (50–88)	0.557	75 (50–88)	63 (38–75)
Pain^b^	78 (55–90)	78 (58–90)	68 (45–83)	0.209	68 (58–90)	78 (48–90)
General health^c^	60 ± 18	62 ± 19	55 ± 16	0.102	64 ± 20	57 ± 16
Physical health domain^b,d^	69 (48–85)	69 (50–85)	68 (46–85)	0.557	73 (60–86)	66 (48–85)
Mental health domain^b,e^	47 (35–73)	52 (35–74)	45 (35–67)	0.404	68 (40–79)	45 (35–64)
Overall HRQOL score^c,f^	60 ± 21	61 ± 22	57 ± 19	0.447	65 ± 21	56 ± 20

CD, celiac disease; GFD, gluten-free diet HRQOL, health-related quality of life; n, number of participants; SF-36, 36-item short form survey

^a^*P* values < 0.050 are considered statically significant

^b^Variables demonstrating skewed distributions are presented as median (interquartile range)

^c^Normally distributed variables are presented as mean ± standard deviation

^d^The average of physical functioning, role limitations due to physical health problems, pain, and general health

^e^The average of role limitations due to emotional problems, energy/fatigue, emotional well-being, and social functioning

^f^The average of physical health domain and mental health domain

### Association between FI, adherence to a GFD, and HRQOL

Table 2 shows the relationship between adherence to a GFD and HRQOL, and between FI and HRQOL. Adherence to a GFD was not significantly associated with any HRQOL scale or domain (*P* > 0.05). Participants facing FI had significantly lower emotional well-being, mental health, and overall HRQOL scores than those with food security (*P* < 0.05). There was a significant association between adherence to a GFD and FI (*P* = 0.02). Most participants not adhering to a GFD were FI (*n* = 21, 81%), while almost half of the participants adhering to a GFD were food secure (*n* = 32, 45%) **(**Table 3).

**Table 3:** Association between food insecurity and adherence to a gluten-free diet

	Non-adherence *n* (%)	Adherence *n* (%)	Total *n* (%)	*P* value
Food Secure	5 (19)	32 (45)	37 (38)	0.020*
Food Insecure	21 (81)	39 (55)	60 (62)	
Total	26 (27)	71 (73)	97 (100)	

n, number of participants

*P values < 0.05 are considered statically significant

## Discussion

This study aimed to assess the influence of adherence to a GFD, FI, and sociodemographic characteristics on HRQOL in patients with CD. Our findings indicated that most participants (73%) were adherent to a GFD, although 62% were experiencing mild to severe FI. Furthermore, FI and age significantly influenced the adherence to a GFD. Participants with low monthly income and those living in non-central regions of Saudi Arabia were more FI and had a poorer HRQOL. FI was significantly associated with both poor adherence to a GFD and poor overall HRQOL. Although adherence to a GFD was not directly associated with HRQOL, FI was significantly associated with HRQOL.

In this study, most of the participants reported FI. Unfortunately, there is no national information about FI in Saudi Arabia; therefore, comparing the result of the present study to the national prevalence of FI was not possible. To our knowledge, few studies have assessed the impact of FI among individuals with CD. In one international study, FI was noted in 42% of 15,819 individuals residing in the Middle East and North Africa—a rate lower than that noted in the presented study (62%) [14]. Previous studies have also indicated that food availability, affordability, and acceptability can influence FI [33]. In our study, participants reported that gluten-free snacks and bread were the two most inaccessible foods in grocery stores in Saudi Arabia. In addition, participants reported several challenges in accessing gluten-free foods in Saudi Arabia, such as high cost and limited availability. These findings are similar to those of a prior study conducted in the UK [16]. Although some studies have indicated that adherence to a GFD may influence FI due to the high cost and limited availability of glute-free foods [14, 16], it is difficult to compare our findings given the dearth of research related to FI among individuals with CD in different countries.

In accordance with previous findings among individuals with CD in the US, Italy, and Brazil [23, 34, 35], most participants in our study adhered to a GFD. Similar to our finding, one previous study noted that 72% of Saudi children with CD adhere to a GFD [36]. However, the adherence to GFD in adults with CD ranged from 30 to 90% in previous studies [8, 9, 37–40]. Such studies have highlighted the potential influence of sociodemographic factors (age, area of residence, age at diagnosis, presence of symptoms during gluten exposure, GFD-related comorbidities, and participation in support groups) on GFD adherence [10–12, 40]. However, in the present study, age was the only sociodemographic factor associated with adherence to a GFD.

In current study, although adherence to a GFD was high, FI was observed in the majority of the participants, and most participants who did not adhere to a GFD showed FI. These findings suggest that FI is highly dependent on adherence to a GFD. Several studies have documented the influence of FI on adherence to a GFD using self-report questionnaires (e.g., Celiac Dietary Adherence Test (CDAT)) and observational methods [41–43]. Other studies have reported the association between FI and adherence to Mediterranean and DASH diets [13, 44, 45]. Low socioeconomic status may also be associated with FI, and therefore poor adherence to the Mediterranean diet [13]. Our findings also indicate that lower income levels may be associated with relatively poorer adherence to a GFD. Several studies have reported that HRQOL is poor among patients with CD [20, 22, 46, 47]. Several factors have been associated with lower HRQOL scores in these patients, including lack of adherence to a GFD, delay in CD diagnosis, and lower education levels [20, 46, 48]. Unsurprisingly, age exhibits a significant negative correlation with physical functioning, which may reflect physical deterioration due to decreased muscle mass and strength [49]. In accordance with previous findings [50], women in our study exhibited lower scores on physical functioning and emotional well-being scales than men. However, medical history (presence of symptoms and age at CD diagnosis) was not significantly associated with HRQOL. This may be because most participants adhered to a GFD. Although, the present study did not find any strong association between BMI and HRQOL possibly due to the small sample size, increased BMI was negatively correlated with physical functioning, as reported in previous studies [50, 51]. Accumulating evidence indicates that there is an association between CD and non-alcoholic fatty liver disease [52], possibly due to the poor nutritional quality of GFD (high in saturated fat, calories, and simple sugars) [53].

In contrast to the current findings, several studies have reported that patients with CD who adhere to a GFD exhibit better HRQOL scores than those who do not [20, 21, 48]. Although this may be because most participants adhered to a GFD, the finding may also be explained by differences in the methods used to assess GFD adherence among studies. Alternatively, the low availability and accessibility of GFD may lead to stress responses such as anxiety and depression, which may explain the association between adherence to a GFD and reduced HRQOL, especially in the emotional health domain. Indeed, previous studies have noted that increased concern related to one’s ability to maintain a food supply can lead to anxiety and depression [14].

To the best of our knowledge, this study is the first to assess the association between FI, adherence to a GFD, and HRQOL in participants with CD living in the Middle East. However, our study possesses some limitations including a small sample size, and the use of self-report questionnaires to assess adherence to a GFD. Serological tests may allow a more objective assessment of GFD adherence [54]. Furthermore, the sensitivity and the specificity of the serology tests for CD diagnosis are not 100%. the present study included participants with confirming CD diagnosed either through a biopsy and serology (n = 24) or based on serology (n = 73). For patients with CD diagnosed through serology tests, the serology tests may include deaminated gliadin, deamidated gliadin peptide, or tissue transglutaminase and the cutoff values for serology tests were not reported. Additionally, the present study did not include an adequate control (non-CD) group because it is difficult to identify a control group (non-CD) following GFD. Moreover, there are no reference values for HRQOL in the Saudi population. However, the HRQOL scores in the current study was similar to those observed in another study on Saudi Arabian patients with CD [20]. Finally, the study participants were recruited from the membership of Celiac Association in Saudi Arabia and an online support group, thus, the sociodemographic and/or disease characteristics of the study population might not be reflective of the lager population with CD living in Saudi Arabia. Nevertheless, many similar studies have recruited participants from non-profit organizations and patients support organizations in the UK, Canada, Finland, and Sweden [22, 46, 55, 56].

## Conclusions

In conclusion, our findings indicated that while most participants adhered to a GFD, they faced varying degrees of FI. Thus, FI significantly influences the adherence to a GFD. In addition, FI was associated with lower HRQOL in terms of both emotional well-being and mental health. Future studies should assess the adherence to a GFD via serological testing to avoid the influence of over- or under-reporting. Additional studies are required to establish reference values of HRQOL for healthy adults living in Saudi Arabia, as it will allow a more effective comparison among studies.

## Data Availability

The datasets used and/or analysed during the current study available from the corresponding author on reasonable request.

## Abbreviations

CD: Celiac disease
FI: Food insecurity
HRQOL: Health-related quality of life
GFD: Gluten-free diet
FIES-SM: Food Insecurity Experience Scale Survey Module
SAR: Saudi Riyal
CDAT: Celiac Dietary Adherence Test
SD: Standard deviation
BMI: Body mass index
IQR: Interquartile range
SF-36: 36-Item Short Form Survey
